# Complete chloroplast genome sequences of two *Alloteropsis* species (Poaceae) from China

**DOI:** 10.1080/23802359.2020.1867014

**Published:** 2021-02-08

**Authors:** Yang Yang, Xianzhi Zhang, Siyun Chen, Zhenhua Guo, Li Wang

**Affiliations:** a College of Agronomy and Biotechnology, Yunnan Agricultural University, Kunming, People’s Republic of China; b Germplasm Bank of Wild Species, Kunming Institute of Botany, Chinese Academy of Sciences, Kunming, People’s Republic of China; c College of Horticulture and Landscape Architecture, Zhongkai University of Agriculture and Engineering, Guangzhou, China

**Keywords:** *Alloteropsis*, chloroplast genome, C4 photosynthesis, phylogenomics

## Abstract

*Alloteropsis* is a widely-distributed genus with C_3_ and C_4_ photosynthetic species belonging to the Poaceae family. This study characterized the complete chloroplast genomes of two *Alloteropsis* species from Chinese mainland, i.e. *Alloteropsis semialata* with C_4_ photosynthetic type and *Alloteropsis cimicina* with C_3_ photosynthetic type. The chloroplast genomes of *A. semialata* and *A. cimicina* are 138,188 bp and 139,335 bp in length, with 38.48% and 38.59% GC contents, respectively. These two chloroplast genomes contain the same number of genes, i.e. 107 unique genes including 27 tRNA, 4 rRNA, and 76 protein-coding genes. Phylogenomic analysis confirmed the species identity of *A. semialata* and *A. cimicina* and supported a close relationship of *Alloteropsis* with species of *Setaria* and *Panicum* in grass family.

*Alloteropsis* (Poaceae: Paniceae) is a genus that comprises approximately 5 species, including C_3_ and C_4_ photosynthetic types (Hattersley and Watson 1992). This genus has a wide geographical distribution, occurring in tropical and Southern Africa, India, SE Asia and Australia. There are two *Alloteropsis* species in China, i.e. *A. semialata* (R. Brown) Hitchcock and *A. cimicina* (Linnaeus) Stapf (Chen and Phillips 2006). *Alloteropsis semialata* is the only known grass which has both C_3_ and C_4_ photosynthetic forms (Ibrahim et al. 2009) while its sister species *A. cimicina* is C_3_ plant. In 1974, Ellis firstly reported that there were two different anatomical structures within *A. semialata,* i.e. ‘Kranz anatomy’ structure and ‘non-Kranz anatomy’ structure (Ellis 1974). According to the leaf anatomical structure and the different photosynthetic types, this species has been divided into two subspecies: *A. semialata* subsp. *semialata* (C_4_ type) and *A. semialata* subsp. *eckloniana* (C_3_ type) (Russell 1983). So far, according to our survey, only C_4_ photosynthetic type *A. semialata* is collected in China and the other species of the same genus, *A. cimicina* has C_3_ photosynthetic type. *Alloteropsis* has been an intriguing model to study the evolution of C_4_ photosynthesis. However, those are insufficient genomic data for molecular study of this genus, compared to other cereal crops such as maize and sorghum (Wang et al. 2009). On this account, this study generated the complete chloroplast genomes of *A. semialata* and *A. cimicina* from Chinese mainland, and it has complemented the data of *Alloteropsis* species in Chinese mainland and coupled with the latest assembly and annotation software.

Young, fresh, and healthy leaves were collected from *A. semialata* in Yunnan (26°27′38″N, 99°53′43″E) and *A. cimicina* in Hainan (19°29′58″N, 110°13′54″E). Both voucher specimens were deposited in the herbarium of Kunming Institute of Botany, Chinese Academy of Science (KUN) with accession numbers of YY3-JC-20 and YY14-Ac-HN-4, respectively. Genomic DNA was extracted via CTAB method (Doyle 1987), then prepared and sequenced on the Illumina Hiseq 4000 platform. About five Gb paired-end data were produced for each species and the data were assembled using GetOrganelle (Jin et al. 2020). To determine the accuracy, we mapped the reads to the result of GetOrganelle in Geneious version 9.1.4 (Kearse et al. 2012). The assembled cp genome was annotated using PGA (Qu et al. 2019), coupled with manual check and adjustment. The ecotype RCH20 of *A. cimicina* (NC_027952) was used as reference for assembling and annotation.

The complete chloroplast genome sequence of *A. semialata* (GenBank accession number MT950759) is 138,188 bp in length. The large single-copy (LSC) and small single-copy (SSC) regions are 81,946 bp and 12,618 bp, which are separated by a pair of inverted repeats (IRs) with 21,812 bp for each. The GC content of whole genome is 38.48%. This genome contains 107 unique genes, including 76 protein-coding genes, 27 tRNA genes, and 4 rRNA genes.

The complete chloroplast genome sequence of *A. cimicina* (GenBank accession number MT950760) is 139,335 bp in length. The lengths of LSC, SSC and IR are 81,747 bp, 12,688 bp and 22,450 bp, respectively. The genome GC content is 38.59%. There are also 107 unique genes in this genome, including 76 protein-coding genes, 27 tRNA genes, and 4 rRNA genes.

To confirm the phylogenetic location of *A. semialata* and *A. cimicina* within the family of Poaceae, a total of 15 chloroplast genomes from Poaceae were analyzed. RAxML–HPC BlackBox (Stamatakis 2014) was used for estimating the maximum likelihood (ML) tree through Cipres Science Gateway (Miller et al. 2010). The result (Figure 1) confirmed the species identity of *A. semialata* and *A. cimicina* samples in this study, which clustered to C_3_ ecotype of *A. angusta* and RCH20 ecotype *A. cimicina* with high supported values (100%), respectively. A close relationship among *Alloteropsis*, *Setaria* and *Panicum* was supported too. These newly sequenced chloroplast genomes of *Alloteropsis* would facilitate to study of the evolution of C_3_ and C_4_ photosynthetic forms in the genus *Alloteropsis*.

**Figure 1.**
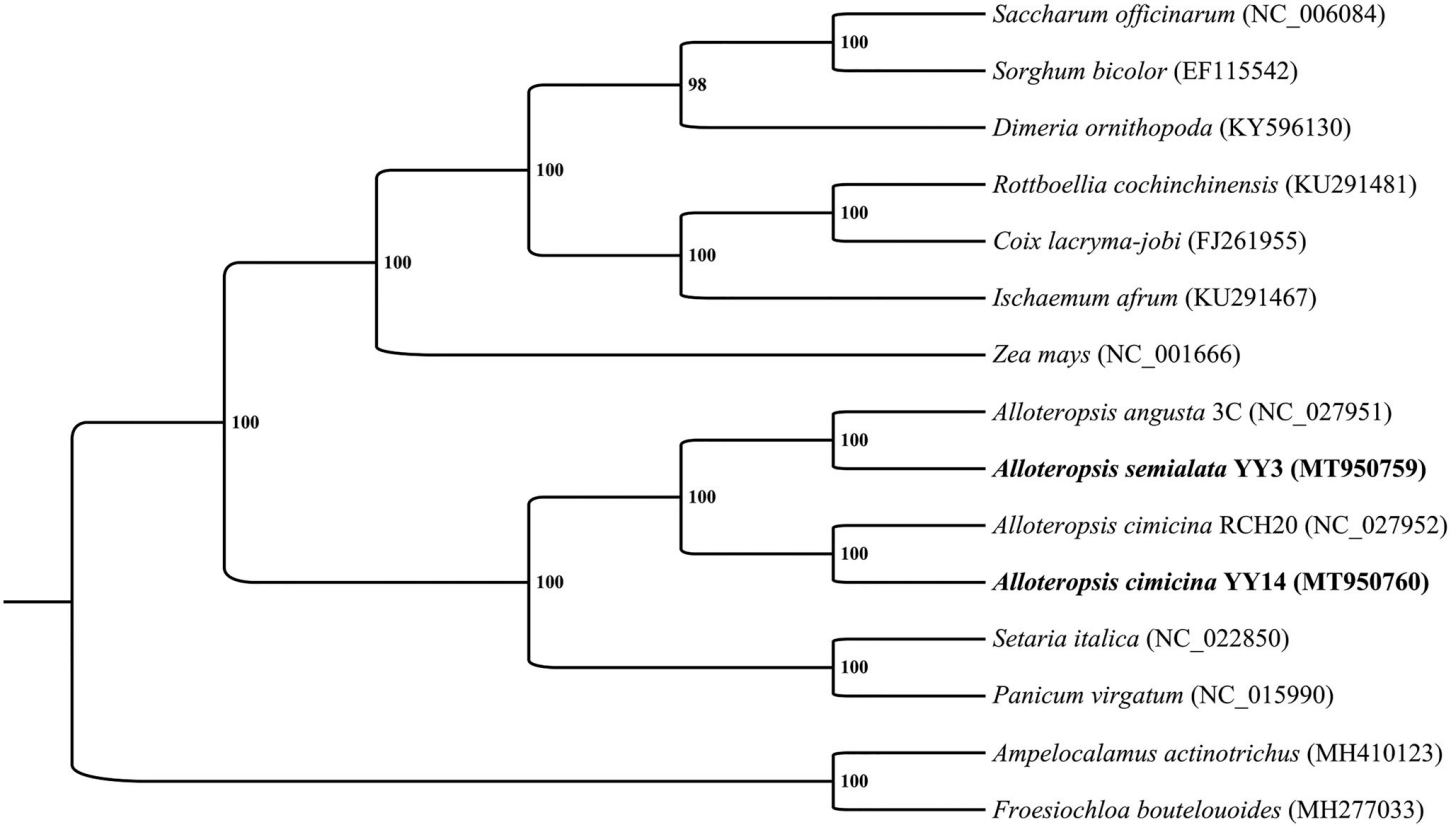
Phylogenetic tree produced by maximum likelihood (ML) analysis base on 15 chloroplast genome sequences from Poaceae, including the newly sequenced *A. semialata* and *A. cimicina* and their closely-related species (the remaining 13 chloroplast genome sequences were download from NCBI, and the GenBank Number is in the parentheses behide every species name, respectively). YY3 is the abbreviation for accession numbers of YY3-JC-20, and YY14 is the abbreviation for accession numbers of YY14-Ac-HN-4. Numbers associated with branched are assessed by Maximum Likelihood bootstrap support values.

## Data Availability

The genome sequence data that support the findings of this study are openly available in GenBank of NCBI at (https://www.ncbi.nlm.nih.gov/) under the accession no. MT950759- MT950760. The associated’BioProject’, ‘SRA’ and ‘Bio-Sample’ numbers are PRJNA672776- PRJNA672775, SRR12927465- SRR12927463, and SAMN16576581- SAMN16576580 respectively.
